# Impact of Air–Sea Turbulent Heat Flux on Eddy-Induced Sea Surface Temperature in the Northwestern Pacific Ocean

**DOI:** 10.3390/s26154665

**Published:** 2026-07-23

**Authors:** Xiangyu Yao, Yunlong Shi

**Affiliations:** 1 State Key Laboratory of Climate System Prediction and Risk Management, Nanjing University of Information Science and Technology, Nanjing 210044, China; yxy@nuist.edu.cn; 2 School of Marine Sciences, Nanjing University of Information Science and Technology, Nanjing 210044, China

**Keywords:** mesoscale eddy, SSTA, air–sea turbulent heat flux, SSTA damping, wind speed

## Abstract

**Highlights:**

**What are the main findings?**

- Eddy-induced SSTA patterns show marked regional and seasonal differences between the STCC and KE regions.
- The STCC exhibits stronger dipole SSTA patterns and higher normalized SSTA damping rates than the KE.

**What are the implications of the main findings?**

- Spatial variations in the normalized damping rate are closely associated with turbulent heat flux response.
- Background wind speed may contribute to spatial variations in the estimated SSTA damping rate within the bulk turbulent heat flux framework.

**Abstract:**

Mesoscale eddies play an important role in upper-ocean heat redistribution, yet the mechanisms controlling eddy-induced sea surface temperature anomaly (SSTA) patterns remain incompletely understood. In this study, we investigate how air–sea turbulent heat flux damping modulates eddy-induced SSTA patterns in the Subtropical Countercurrent (STCC) and Kuroshio Extension (KE) regions of the northwestern Pacific. Using satellite observations, reanalysis products, and eddy trajectory data from 2010 to 2019, we composite cyclonic and anticyclonic eddies in different seasons and quantify the relative contributions of monopole and dipole SSTA components. The results show that the STCC region exhibits a larger dipole contribution and a higher normalized SSTA damping rate than the KE region in both warm and cold seasons. This regional contrast suggests that stronger SSTA damping is associated with a more pronounced dipole SSTA pattern, whereas weaker damping favors a more monopole structure. The spatial distribution of the normalized damping rate closely resembles that of the turbulent heat flux response rate, while mixed-layer depth appears to play a secondary role in shaping the large-scale damping pattern. In addition, the damping rate increases with background wind speed, indicating that wind speed may modulate eddy-induced SSTA patterns by enhancing turbulent heat flux feedback. These findings highlight the potential role of air–sea turbulent heat flux damping in shaping regional differences in eddy-induced SSTA patterns and provide a useful perspective for understanding mesoscale air–sea interaction in the northwestern Pacific.

## 1. Introduction

Mesoscale eddies are ubiquitous features of the ocean that represent an indispensable component of oceanographic research. With horizontal-length scales ranging from tens to hundreds of kilometers, and temporal scales spanning tens to hundreds of days, influencing hundreds of meters or even deeper water layers, eddies play a significant role in oceanic material transport, redistribution of salt and heat over the oceans, and biogeochemical processes [1,2,3,4,5,6]. Mesoscale eddies are also one of the primary sources of sea surface temperature (SST) variability, and SST anomaly (SSTA) is a crucial indicator reflecting the dynamic characteristics of mesoscale eddies, capable of characterizing their dynamic and thermodynamic structures.

Meanwhile, the sea surface temperature anomaly (SSTA) associated with oceanic mesoscale eddies exerts profound impacts on both oceanic dynamics and atmospheric circulation systems [7,8,9,10]. The SSTA caused by eddies can affect the spatial distribution and intensity of the wind field above them, effectively building a dynamic bridge that allows energy to be input into the ocean interior [7]. Warm-core eddies characterized by positive SSTA at the eddy center can cause anomalies in turbulent heat flux, leading to a significant deepening of the atmospheric boundary layer [8]. In addition, the forcing of eddy-induced SST can trigger an increase in local coastal precipitation [9]. Therefore, in-depth research on the formation, evolution, and potential regulatory mechanisms of eddy-induced SSTA can not only enhance our understanding of the mesoscale ocean dynamic energy cycle and thermal processes but also help to reveal the key role of mesoscale ocean processes in the air–sea interaction system.

The diversity in SSTA patterns was supposed to be one of the indications of the key process through which eddies drive SSTA. Over recent decades, advances in satellite remote sensing have yielded vast long-term, global-scale observational datasets, with satellite altimetry offering unique advantages in temporal resolution and spatial coverage. These developments have provided reliable data support for large-sample mesoscale eddy identification, tracking, and detection, enabling systematic characterization of eddy-induced SSTA patterns across the ocean [11,12,13]. Existing studies have revealed that the eddy-induced SSTA has two typical patterns: the monopole pattern, with only a single positive or negative SSTA extreme value at the eddy center; and the dipole pattern, where two SSTA extrema of opposite signs are aligned along the eddy propagation direction [14,15].

The SSTA pattern has significant regional heterogeneity [16,17]. In general, the spatial distribution of eddy-induced SSTA patterns is tightly coupled to regional eddy kinetic energy levels. In regions with high eddy kinetic energy, such as western boundary current systems and the Southern Ocean, the SSTA displays warm/cold monopoles for anticyclones/cyclones, while the regions with low eddy kinetic energy tend to have a dipole pattern [17]. Many studies have attributed the pattern of eddy-induced SSTA to the eddy-stirring effect, which causes a dipole pattern [14,16,18], and vertical pumping, which causes a monopole pattern [14,17]. However, some researchers have found that the air–sea turbulent heat flux also plays a significant role in regulating the eddy-induced SSTA [13,19,20,21,22,23], and clarifying the relationship between air–sea turbulent heat flux and sea surface temperature has become a research hotspot in the field of air–sea interaction [24,25,26]. Sophia Moreton et al. found that the temporal and spatial variation in SSTA are mainly determined by the air–sea turbulent heat flux [19].

Air–sea turbulent heat flux exhibits a well-defined response to SSTA known as turbulent heat flux feedback, a fundamental process in air–sea coupling systems [27] and a key metric for quantifying SSTA damping rates [19]. Within this framework, the magnitude of turbulent heat flux response is defined as the variation in surface turbulent heat flux per 1 K change in SSTA [19,28]. There is a strong linear positive correlation between the eddy-related heat flux and SSTA, which means a positive SSTA corresponds to a positive anomaly of the air–sea turbulent heat flux, while a negative SSTA corresponds to a negative anomaly of the air–sea turbulent heat flux [22,23]. Furthermore, the amplitude of eddy-induced SSTA is positively correlated with the magnitude of the accompanying turbulent heat flux anomaly, with larger SSTA amplitudes corresponding to stronger turbulent heat flux anomalies [23]. By altering the thermal stability of the sea surface, eddy-induced SSTA can also modulate the intensity of turbulent heat flux [22].

This feedback relationship directly governs the variation in eddy-induced SSTA: the SSTA of mesoscale eddies will trigger abnormal air–sea turbulent heat flux, which will suppress SSTA; that is, produce a damping effect on SSTA [21,28]. The SSTA damping parameterizes the attenuation of SSTA to study the influence of air–sea turbulent heat flux shaping on the eddy-induced SSTA pattern. Enhanced SSTA damping will transform the SSTA pattern from a monopole to a dipole and reduce its amplitude at the same time. Globally, the SSTA damping increases from the poles towards the equator [21]. Therefore, in the mid- and high-latitude regions (such as the Kuroshio, the Gulf Stream, and the Southern Ocean), the SSTA damping is small, and the SSTA pattern shows a monopole pattern with a large amplitude; while in the tropical and subtropical regions, the damping is high, and the SSTA is mainly in a dipole pattern with a small amplitude.

Notably, previous studies have mainly focused on the distribution characteristics of the damping of eddy-induced SSTA on a global scale, without discussing seasonal variation patterns and influencing mechanisms of the damping rate. Our study focuses on analyzing the distribution of the damping rate in specific regions and the seasonal variation characteristics. The northwestern Pacific is a critical region for heat, salt, and energy exchange between the equatorial and subtropical Pacific, playing a disproportionately important role in global and regional climate [29,30,31]. It is also a hotspot of mesoscale eddy activity, with numerous long-lived eddies generated annually [32,33,34]. Therefore, in this study, the Subtropical Countercurrent (STCC) and the Kuroshio Extension (KE) are selected as the study regions, and a year is divided into the cold season (from November to April) and the warm season (from May to October). The seasonal variation characteristics of SSTA damping and its regulatory effect on the eddy-induced SSTA pattern are investigated. By analyzing the influencing factors of the SSTA damping, its driving mechanism is further revealed.

The remaining parts of this paper are organized as follows: Section 2 outlines the data and methods used in our analysis. Section 3 presents the seasonal differences in composite eddy-induced SSTA patterns across the two study regions, analyzes the seasonal variation in SSTA damping rate and its regulatory effect on the eddy-induced SSTA pattern, and discusses the mechanisms linking background environmental conditions to damping rate variability. Section 4 summarizes the main conclusions of this study and highlights prospects for future research.

## 2. Materials and Methods

### 2.1. Data

#### 2.1.1. Eddy Dataset

The eddy dataset used in this study is the 4th-edition eddy dataset from earlier eddy products [12]. This version of the eddy dataset is produced by the Collecte Localisation Satellites/Data Unification and Altimeter Combination System (CLS/DUACS) group and released by the Archiving, Validation and Interpretation of Satellite Oceanographic Data (AVISO) platform. Its identification and tracking of eddies are based on the sea-surface height anomalies measured by AVISO satellite altimeters over many years. Eddies are defined by connected pixels that meet specific criteria, and the dataset includes information on the time, longitude, latitude, amplitude, radius, polarity, and trajectory of eddies. To a large extent, this dataset reduces the number of “erroneously” detected eddies, with a temporal resolution of 1 day. Due to its accuracy, it has been widely used in global ocean eddy research [35]. The data used in this paper cover the period from 2010 to 2019.

#### 2.1.2. Air–Sea-Related Data

The air–sea-related data used in this study come from the monthly average dataset of the ERA5 reanalysis dataset. This global atmospheric reanalysis dataset is produced by the European Centre for Medium-Range Weather Forecasts (ECMWF), whose model incorporates observational inputs from a wide range of platforms, including both satellite retrievals and in situ ground-based measurements [36]. It can be traced back to 1940 and has replaced the ERA-Interim reanalysis dataset. Reanalysis data are not restricted by timely forecast release. When tracing more distant historical data, improved versions of the original observational data can be incorporated, greatly improving the quality of the product. The dataset selected in this paper includes information on atmospheric dew-point temperature, sea surface atmospheric pressure, near-surface atmospheric temperature, SST, and sea surface wind speed at 10 m. The sea surface temperature data here is used to calculate the heat flux response rate (Equation (2)). The spatial resolution is 0.25° × 0.25°, and the time range is from 2010 to 2019.

#### 2.1.3. Ocean Mixed-Layer Depth Data

The mixed-layer depth data used in this paper are calculated by James Holte et al. based on Argo profile data using a hybrid algorithm and a standard threshold method [37]. This climate-characteristic data includes nearly 2.62 million Argo profile data up to April 2022, providing accurate information on the characteristics, range, and seasonal variation patterns of the global mixed-layer depth. The mixed-layer depth calculated by the hybrid algorithm used in this data is shallower and usually more accurate than that calculated by the threshold method. In this paper, the monthly average data of the mixed-layer depth are downloaded, with a spatial resolution of 1° × 1°.

#### 2.1.4. Eddy Sea Surface Temperature Data

Here we select the sea surface temperature field from the Operational Sea Surface Temperature and Sea Ice Analysis (OSTIA) system. This data can be downloaded from CMEMS, and the product ID is SST_GLO_SST_L4_REP_OBSERVATIONS_010_011. This product adopts the optimal interpolation algorithm, which utilizes spatial autocorrelation and temporal consistency to produce a high-resolution gridded SST product. It has a spatial resolution of 0.05° × 0.05° and a daily temporal resolution, covering the period from 1 October 1981 to 31 December 2020. Spanning more than 30 years, the dataset comprises over 14,000 daily records in total. In this study, we use the subset from 2010 to 2019 for the investigation of eddy-induced SST variability.

### 2.2. Model and Method

#### 2.2.1. Estimation of Eddy-Induced SSTA Damping Rate

In this study, we investigate the influence of air–sea turbulent heat flux on eddy-induced sea surface temperature anomaly (SSTA), adopting a two-dimensional SSTA stir-ing-damping model [21]. In this model, the air–sea turbulent heat flux induced by ocean eddies is parameterized as $\lambda T^{\prime }$ that weakens SSTA, where λ represents the SSTA damping rate and $T^{\prime }$ represents the SSTA.

The SSTA damping rate λ, a core physical quantity throughout this study, can be calculated through the sensible heat and latent heat response rates $\alpha_{s}$ and $\alpha_{L}$ [21]:

(1) $$\lambda =\frac{\alpha_{s}+\alpha_{L}}{\rho_{0}c_{p}H}$$

where $\rho_{0}$ is the sea water density, $c_{p}$ is the specific heat capacity of sea water, and H is the mixed-layer depth. In this study, $\rho_{0}$ is assumed constant at 1025 kg m^−3^, and $c_{p}$ is fixed at 4000 J kg^−1^ K^−1^, and the resulting unit of λ is day^−1^.

The sensible heat and latent heat response rates are physically defined as the partial derivative of turbulent heat flux anomaly against SSTA [21]. Previous studies have shown that sensible and latent heat fluxes $Q_{s}$ and $Q_{L}$ can be expressed using bulk formulas as [38]:

(2) $$Q_{s}=\rho_{air}c_{a}c_{h}u_{a}(SST-T_{air})$$

(3) $$Q_{L}=\rho_{air}L_{e}c_{e}u_{a}(q_{sat}-q_{a})$$

here $\rho_{air}$ is the air density, $c_{a}$ is the specific heat capacity, $c_{h}$ is the transfer coefficient of sensible heat flux, $u_{a}$ is the 10 m wind speed, $T_{air}$ is the air temperature, $L_{e}$ is the latent heat of evaporation, $c_{e}$ is the transfer coefficient for latent heat flux, $q_{sat}$ is the interfacial value of the water vapor mixing ratio that is computed from the saturation mixing ratio for pure water at the SST, $q_{a}$ is the specific humidity of the surface air. $\rho_{air}$ is calculated from dew-point temperature and atmospheric pressure based on Dalton’s law of partial pressures, and $q_{sat}$ is calculated via the Clausius–Clapeyron equation. $c_{h}$ and $c_{e}$ are parameterized as functions of saturation specific humidity $q_{sat}$, air–sea temperature difference and 10 m wind speed $u_{a}$. Following previous studies [21], the constants are set to $c_{a}=1004$ J kg^−1^ K^−1^ and $L_{e}=2.5\times 10^{6}$ J kg^−1^ for the domain between 60° S and 60° N.

Therefore, the response rates are approximated as:

(4) $$\alpha_{s}=\frac{dQ_{s}^{\prime }}{dT^{\prime }}=\rho_{a}c_{a}c_{h}u_{a}$$

(5) $$\alpha_{L}=\frac{dQ_{L}^{\prime }}{dT^{\prime }}=\rho_{a}L_{e}c_{e}u_{a}\frac{dq_{sat}}{dSST}|{SST}_{0}$$

here $Q_{s}^{\prime }$ is the sensible heat flux anomaly, $Q_{L}^{\prime }$ is the latent heat flux anomaly, and ${SST}_{0}$ is the time-averaged SST.

Since the eddy-stirring rate U/2πR modulates the magnitude of the damping rate λ, we normalize the damping rate to obtain the dimensionless form $\lambda^{*}$, a dimensionless quantity [21].

(6) $$\lambda^{*}=\frac{2\pi R\lambda }{U}$$

here U is the azimuthal speed of the eddies and R is the eddy radius.

#### 2.2.2. Composed Eddy-Induced SSTA

Eddy-induced SSTA is computed as the anomaly of daily SST with respect to the climatological mean SST. All data grid points (X, Y) of the SSTA field within a distance of two times the eddy radius from the eddy center are selected, where the zonal and meridional coordinate differences have been pre-converted into geophysical distance units. The selected points are finally normalized by the radius of the corresponding eddy:

(7) $$X=\frac{\left({Lon}_{SST}-{Lon}_{e}\right)}{R_{e}}$$

(8) $$Y=\frac{\left({Lat}_{SST}-{Lat}_{e}\right)}{R_{e}}$$

here ${Lon}_{SST}$ and ${Lat}_{SST}$ are the longitude and latitude of the SST, ${Lon}_{e}$ and ${Lat}_{e}$ are the longitude and latitude of the eddy center, $R_{e}$ is the radius of the corresponding eddy. After normalization, the transformed coordinates X and Y satisfy the constraint −2 ≤ *X*, *Y* ≤ 2. Each eddy-matched SSTA field is then interpolated onto a uniform 0.25 × 0.25 grid. Finally, the composite pattern of eddy-induced SSTA is obtained by calculating the domain-averaged value of the normalized eddy-induced SSTA for different sea regions.

#### 2.2.3. Pattern of Eddy-Induced SSTA

The SSTA pattern caused by mesoscale eddies contains monopole and dipole components. To quantify the similarity between the observed SSTA and the monopole–dipole pattern, we divide the eddy-induced SSTA into two parts: the monopole and the dipole. The monopole component is derived by averaging the SSTA pattern of the composite eddy along the radial direction, and the residual corresponds to the dipole component. We use the method proposed by Frenger et al. [39] to calculate the contribution rates of the two parts to the entire eddy-induced SSTA:

(9) $$V_{total}=\sum_{i=1}^{n}{\left[SSTA{\left(total\right)}_{i}\right]}^{2}$$

(10) $$V_{m}=\sum_{i=1}^{n}{\left[SSTA{\left(monopole\right)}_{i}\right]}^{2}$$

(11) $$P_{m}=\frac{V_{m}}{V_{total}}$$

(12) $$P_{d}=1-P_{m}$$

here $V_{total}$ denotes the total variance of eddy-induced SSTA from the eddy composite; $V_{m}$ represents the variance of the decomposed monopole component of eddy-induced SSTA. $P_{m}$ and $P_{d}$ are the respective contributions of the monopole and dipole patterns relative to the total SSTA pattern. n is the number of pixels in the eddy composite within one eddy radius from the eddy center.

All the visualization and numerical plotting in this work are performed using MATLAB R2024b (MathWorks Inc., Natick, MA, USA).

## 3. Results

### 3.1. The SSTA Pattern of Mesoscale Eddies

**Figure 1:**
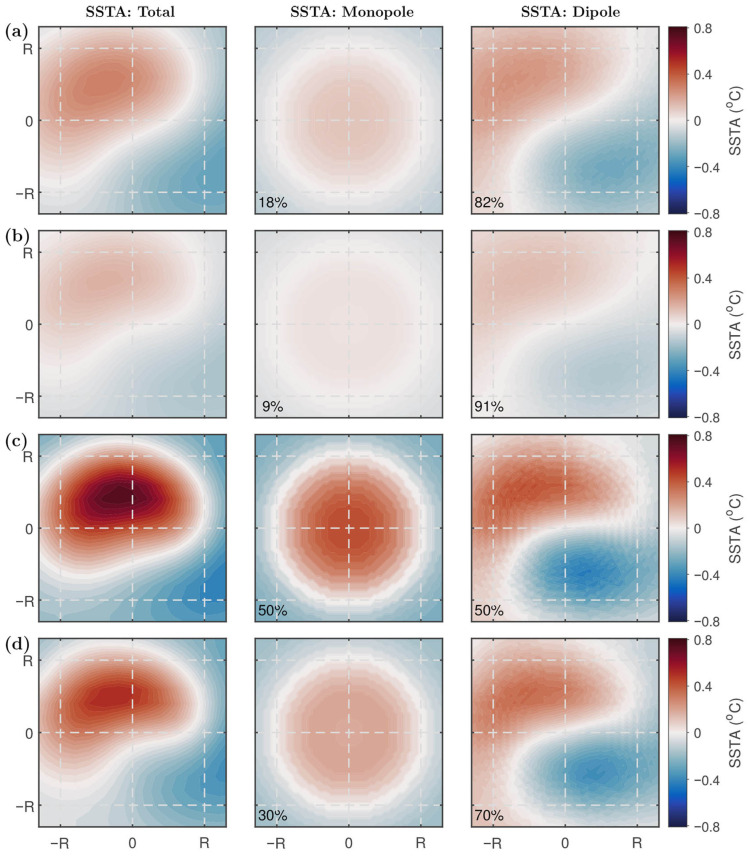
Seasonally composite pattern of anticyclonic sea surface temperature anomaly (SSTA) and its decomposed components in the Subtropical Countercurrent (STCC) and the Kuroshio Extension (KE) regions. The first column is the total SSTA pattern, the second column is the monopole component, and the third column is the dipole component. (**a**) SSTA in the STCC region during cold season; (**b**) SSTA in the STCC region during warm season; (**c**) SSTA in the KE region during cold season; (**d**) SSTA in the KE region during warm season.

In this section, we explore the SSTA patterns of eddies in the Subtropical Countercurrent (STCC) and the Kuroshio Extension (KE) regions during cold and warm seasons. Figure 1 shows the SSTA patterns of anticyclonic eddies. As shown in the first column of Figure 1, in both study regions, positive SSTA associated with anticyclonic eddies are located in the northwestern part of the eddies, while negative anomalies reside in the southeastern part, forming a distinct dipole pattern. To further quantify the eddy-induced SSTA composition, the contribution rates of the monopole and dipole components were calculated (Equations (7)–(10)), with the results presented in the second and third columns of Figure 1.

The result indicates that in the STCC region, the dipole component accounts for 82% of the total SSTA variance in the cold season and 91% in the warm season, which is substantially larger than that of the monopole component, demonstrating that the SSTA of anticyclonic eddies in the STCC is predominantly characterized by a dipole pattern. For the KE region, the dipole contribution is 50% in the cold season, lower than the 70% in the warm season. In this region, the dipole component dominates over the monopole component during the warm season, leading to a pronounced SSTA dipole pattern, whereas during the cold season, the monopole and dipole components contribute equally, suggesting that the cold-season SSTA pattern is jointly controlled by both modes. Furthermore, for both the total eddy-induced SSTA field and the individual monopole/dipole components, the SSTA amplitude in the KE region is significantly larger than that in the STCC region. Additionally, both regions exhibit a consistent seasonal feature: the SSTA amplitude is higher in the cold season than that in the warm season.

**Figure 2:**
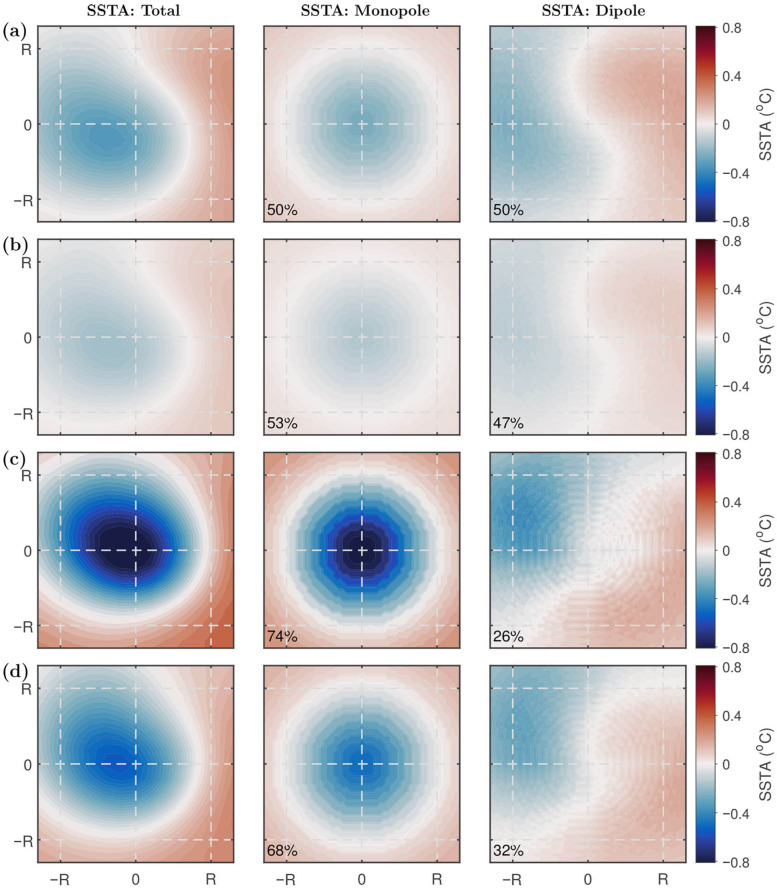
Seasonally composite pattern of cyclonic SSTA and its decomposed components in the STCC and KE regions. The first column is the total SSTA pattern, the second column is the monopole component, and the third column is the dipole component. (**a**) SSTA in the STCC region during cold season; (**b**) SSTA in the STCC region during warm season; (**c**) SSTA in the KE region during cold season; (**d**) SSTA in the KE region during warm season.

As shown in Figure 2, cyclonic eddies in both the STCC and KE regions generally exhibit a monopole pattern with negative anomalies centered on the eddy core. Consistent with the analysis for anticyclonic eddies, we also calculated the contribution rates of the monopole and dipole components for cyclonic eddy-induced SSTA (second and third columns of Figure 2).

The result reveals that the SSTA pattern of cyclonic eddies also has significant regional and seasonal differences between cold and warm seasons. In terms of the dipole contribution, in the KE region, the dipole pattern is weakest in the cold season, accounting for only 26% of the total SSTA, and only increases to 32% in the warm season. This confirms that cyclonic eddy-induced SSTA in the KE region is dominated by the monopole pattern. In contrast, the dipole pattern is more pronounced in the STCC region, contributing 50% in the cold season and 47% in the warm season, indicating that cyclonic eddy-induced SSTA in this region is co-dominated by both the monopole and dipole components. In addition, similar to the results for anticyclonic eddies, the SSTA amplitude of cyclonic eddies in the KE region is significantly larger than that in the STCC region, and the amplitude in both regions is higher in the cold season than in the warm season.

In summary, the SSTA patterns of both anticyclonic and cyclonic eddies exhibit clear spatio-temporal differences. The contribution rate of the dipole component to eddy-induced SSTA in the KE region is consistently lower than that in the STCC region. In general, the dipole contribution is higher in the warm season, with the only exception of cyclonic eddies in the STCC region. The SSTA amplitude of eddies in both regions is higher in the cold season than in the warm season. Across all regions and seasons, the dipole contribution of anticyclonic eddies is always higher than that for cyclonic eddies. Anticyclonic eddies are predominantly characterized by a dipole pattern, while cyclonic eddies tend to be dominated by a monopole pattern.

### 3.2. Temporal and Spatial Distribution of SSTA Damping Rate

The two-dimensional stirring-damping model links the observed variations in eddy-induced SSTA patterns to the damping rate λ [21]. In this section, we will investigate the spatio-temporal variations in λ.

**Figure 3:**
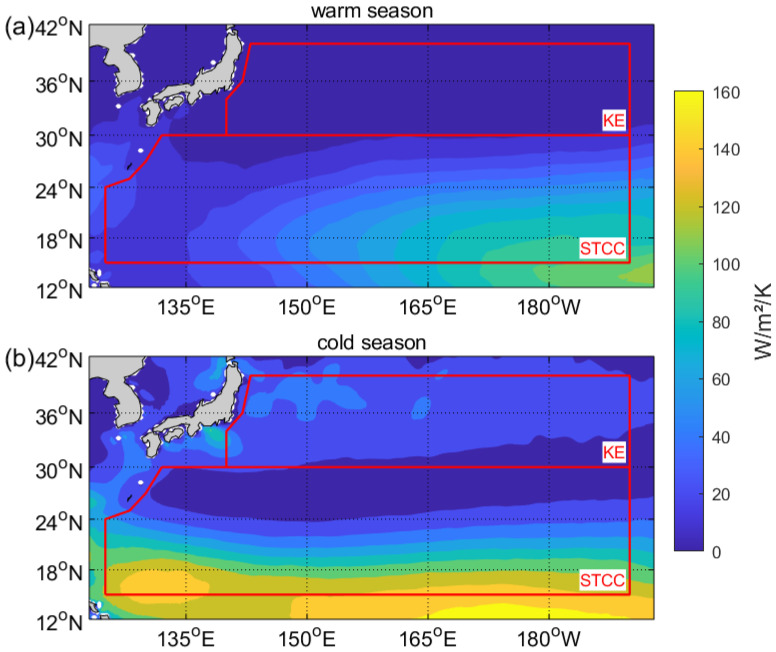
Spatial distributions of heat flux response rate in the western North Pacific: (**a**) Warm season; (**b**) cold season. The red boxes mark the KE and STCC regions. Color bars show units: W m^−2^ K^−1^.

Figure 3 shows the spatio-temporal distribution patterns of the air–sea turbulent heat flux response rate, defined as the sum of the latent heat response rate and the sensible heat response rate. The result shows that the heat flux response rate exhibits a distinct zonal gradient, with higher values in low latitudes and lower values in higher latitudes. Specifically, the mean heat flux response rate in the STCC region is higher than that in the KE region. A further comparison reveals that in the cold season, there is a high-value area in the northeastern part of the KE region, with a value reaching up to 40 W m^−2^ K^−1^, while in the warm season, the heat flux response rate is relatively small, generally below 20 W m^−2^ K^−1^. In contrast, the STCC region displays a clear east–west contrast in the warm season, characterized by a west-low–east-high gradient. Regarding seasonal variation, the response rate isopleths during the cold season are generally oriented zonally. In the STCC region, although the maximum response rate in the cold season exceeds that in the warm season, the area with response rates higher than 20 W m^−2^ K^−1^ is relatively confined, located mostly south of 24° N. But in the warm season, by contrast, this high-value region extends northward to the northern boundary of the entire study domain.

**Figure 4:**
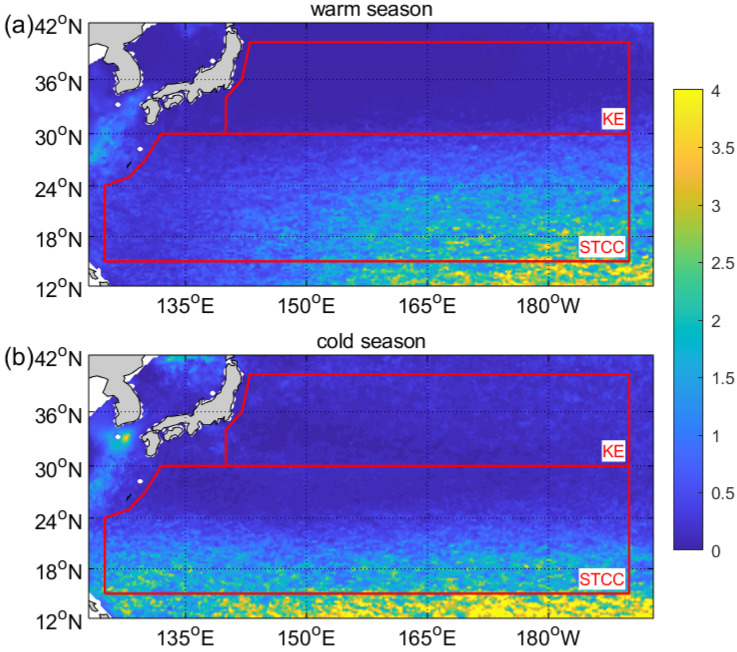
Spatial distributions of normalized SSTA damping rate in the western North Pacific: (**a**) Warm season; (**b**) cold season. The red boxes mark the KE and STCC regions.

The results of the damping rate distribution (Figure 4a,b) reveal that, similar to the heat flux response rate, whether in the warm season or the cold season, the distribution of $\lambda^{*}$ shows a feature typical of zonal distribution in that it decreases from low latitudes to high latitudes. By further comparing the spatial changes between the warm and cold seasons, it can be found that the values in the KE region are generally low. And in the northern region, the values in the warm season are slightly lower than those in the cold season, while in the southern region, the values in the warm season are slightly higher than those in the cold season. In addition, the seasonal variation in the spatial distribution of $\lambda^{*}$ in the STCC region is significant. Although the overall decreasing trend from low latitudes to high latitudes is still maintained, there are obvious east–west differences in the warm season, showing an overall decreasing trend from the southeast to the northwest, which has a good corresponding relationship with the distribution of the heat flux response rate. Taking 150° E as the boundary, $\lambda^{*}$ in the northeastern part of this region is higher in the warm season than in the cold season, while in the southwestern part, it is higher in the cold season than in the warm season.

**Figure 5:**
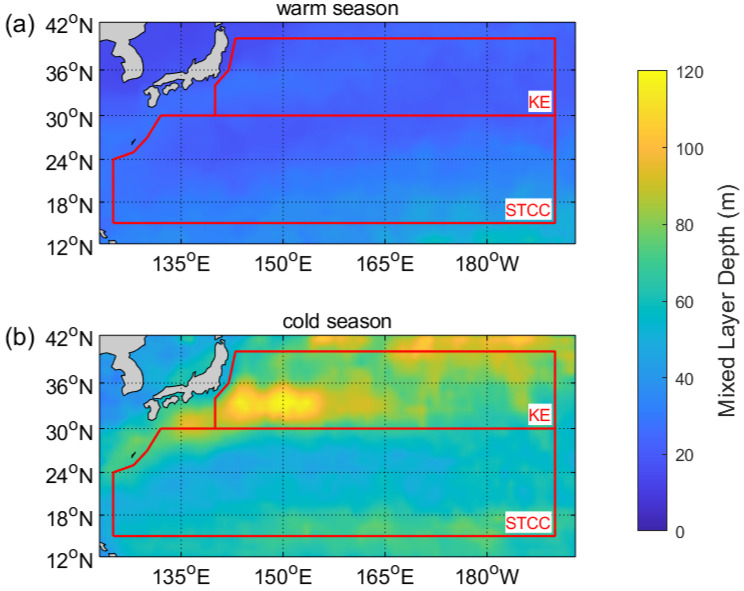
Spatial distribution of mixed-layer depth in the western North Pacific: (**a**) Warm season; (**b**) cold season. The red boxes mark the KE and STCC regions. Color bars show units: m.

It can be seen from Figure 5 that the overall values of the mixed-layer depth in the STCC and KE regions are higher in the cold season than in the warm season. In the warm season, the mixed-layer depth in the entire region is generally less than 40 m. Only in some regions of the KE does the depth slightly increase to about 50 m. In the southern STCC region, the depth is generally maintained within 30 m, there is no obvious large-scale deep mixed-layer area, and the overall spatial gradient is weak. In the cold season, the mixed-layer depth in the northern KE region deepens significantly. In most regions, the depth reaches 60–100 m, and in some regions, it exceeds 100 m. Although the mixed-layer depth in the southern STCC region increases compared with that in the warm season, it is still generally maintained in the range of 40–60 m, which is shallower than that in the northern KE region.

According to the calculation formula of the SSTA damping rate, the magnitude and spatial distribution of the SSTA damping rate are jointly determined by two key factors: the air–sea heat flux response rate and the mixed-layer depth. Comparing the spatial distribution patterns of the SSTA damping rate, the heat flux response rate, and the mixed-layer depth, the overall spatial structure of the SSTA damping rate is highly consistent with the heat flux response rate. Both show a significant increasing trend from the high-latitude KE region to the low-latitude STCC region, and the spatial changes in the warm and cold seasons are consistent. In the warm season, it shows a distribution pattern of being lower in the west and higher in the east, while in the cold season, it is nearly horizontally distributed. In contrast, the mixed-layer depth does not show a similar spatial change, nor does it exhibit the corresponding distribution characteristics as the SSTA damping rate. These results suggest that, in the present study region, the large-scale spatial pattern of the normalized SSTA damping rate is more strongly associated with the turbulent heat flux response rate than with mixed-layer depth.

### 3.3. Mechanism of the Influence of Damping Rate on Eddy-Induced SSTA Pattern

In the previous two sections, the spatio-temporal characteristics of the eddy-induced SSTA pattern and the SSTA damping rate were presented. This section will combine the results to investigate the influence of the SSTA damping rate on the eddy-induced SSTA pattern.

We quantitatively calculate the average normalized damping rate $\lambda^{*}$ of the two regions in warm and cold seasons, respectively, aiming to explore more deeply and systematically the regulatory effect of the damping rate on the spatial pattern of the mesoscale eddy-induced SSTA, and to further clarify the important position of the damping effect in shaping the eddy-induced SSTA pattern. In terms of the overall average damping rate level of the two research regions, the statistical results show that the average $\lambda^{*}$ in the STCC region reaches 0.8706, significantly higher than the average damping rate of 0.1299 in the KE region. There are considerable regional differences in the intensity of the damping rate between the two regions.

To establish a relationship between the damping rate level and the SSTA pattern, we compare the contribution rates of the monopole and dipole components of the eddy-induced SSTA in the two regions. The result shows that the contribution rate of the dipole component of the eddy-induced SSTA in the STCC region is higher than that in the KE region in both warm and cold seasons. This result indicates that compared with the KE region, the anticyclonic eddies in the STCC region are closer to the typical dipole SSTA pattern, while the eddy-induced SSTA in the KE region is more inclined to the monopole pattern characteristics. Thus, the core conclusion can be preliminarily drawn: the larger the standardized damping rate $\lambda^{*}$, the more significant the dipole pattern characteristics of the eddy-induced SSTA; on the contrary, the lower the damping rate, the more obvious the monopole pattern characteristics of the SSTA.

To verify the consistency of this inference under different seasonal backgrounds, we further conduct a verification analysis from the dimension of seasonal changes. The quantitative calculation results by season show that: in the warm season, the average $\lambda^{*}$ in the STCC region is 0.9273, corresponding to 0.0787 in the KE region; while in the cold season, the average $\lambda^{*}$ in the STCC region drops to 0.8139, and the average $\lambda^{*}$ in the KE region is only 0.1812. From these results, it can be clearly seen that whether in the warm or cold season, the average standardized damping rate in the STCC region is stably higher than that in the KE region, and this regional difference does not change with the seasonal transition.

Combined with the statistical results of the spatial pattern of the eddy-induced SSTA, the contribution rate analysis of the corresponding seasons further supports the above conclusion: in the warm season, the contribution rate of the SSTA dipole component of the anticyclonic eddies in the STCC region reaches 91%, significantly higher than 70% of that in the KE region; in the cold season, the contribution rate of the SSTA dipole component of the anticyclonic eddies in the STCC region is 82%, which is also much higher than that of 50% in the KE region; the statistics of the cyclonic eddies also result in consistent conclusions: the contribution rate of the SSTA dipole component of the cyclonic eddies in the STCC region is higher than the corresponding values in the KE region in both the warm and cold seasons. Through the cross-comparison analysis of the average damping rate and the spatial pattern of the eddy-induced SSTA in different seasons and different research regions, the regulatory effect of the damping rate on the SSTA pattern is further confirmed, providing observational evidence in both regional and seasonal dimensions for the universality of this mechanism.

A comparative analysis of warm and cold seasons reveals that the contribution rate of the eddy-induced SSTA dipole component in the warm season is slightly higher than that in the cold season. However, the average SSTA damping rate in the KE region shows a characteristic of being lower in the warm season than in the cold season. This indicates that the damping rate is not the dominant controlling factor for the seasonal variation in the SSTA pattern, and the seasonal differences in this pattern may be jointly regulated by other processes.

### 3.4. Investigation on the Influencing Factors of Damping Rate

Our previous research found that the damping rate of SSTA is consistent with the change in the air–sea turbulent heat flux response rate. To further explore the relationship between them, this study adopts a grouped statistical analysis strategy. Specifically, the heat flux response rates are divided into 13 continuous intervals from 0 to 130, with an interval step of 10. For all eddy samples falling within each interval, we calculate the average value of their normalized damping rates, so as to systematically examine the overall statistical correlation between the two variables. It is worth noting that the sample size of each interval exceeds 1000, which ensures a sufficient sample size and effectively reduces the estimation error of the average value, fully meeting the basic requirements of reliable statistical analysis.

**Figure 6:**
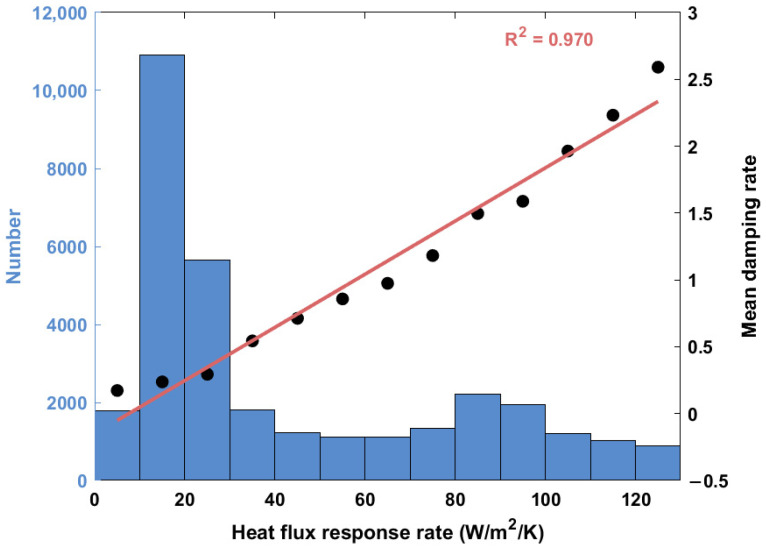
Variation in the domain-averaged normalized damping rate as a function of the heat flux response rate. Background bar plots indicate the number of eddies counted in each bin along the *x*-axis. The red solid line denotes the linear regression fit, and R^2^ indicates the coefficient of determination.

The statistical results presented in Figure 6 clearly demonstrate that there is a significant positive linear correlation between the average normalized damping rate and the heat flux response rate: as the heat flux response rate increases, the corresponding average damping rate rises synchronously, and the coefficient of determination (R^2^) reaches as high as 0.970, indicating an extremely high fitting degree. The result shows that the SSTA damping rate changes synchronously with the heat flux response rate.

Cayan et al. have pointed out that the variation in air–sea heat flux in the Northwest Pacific Ocean is significantly modulated by wind speed [40]. On this basis, through data analysis, Chen et al. further confirmed that the magnitude of both latent heat flux anomaly and sensible heat flux anomaly increases monotonically with the increase in wind speed [41]. The definition formulas of heat flux response rate (Equations (1) and (2)) also directly reflect that the response rate increases with the enhancement of wind speed. Combined with the above conclusion, the normalized damping rate $\lambda^{*}$ is proportional to the heat flux response rate obtained in Figure 5a, it can be logically inferred that the change in the wind speed will generally affect the magnitude of $\lambda^{*}$, which means wind speed can influence eddy-induced SSTA through changing the efficiency of air–sea turbulent heat flux.

Based on this scientific inference, this paper further carries out targeted research on the influence of wind speed on the normalized damping rate of SSTA, aiming to deepen the understanding of the core controlling factors of eddy-induced SSTA spatial pattern.

**Figure 7:**
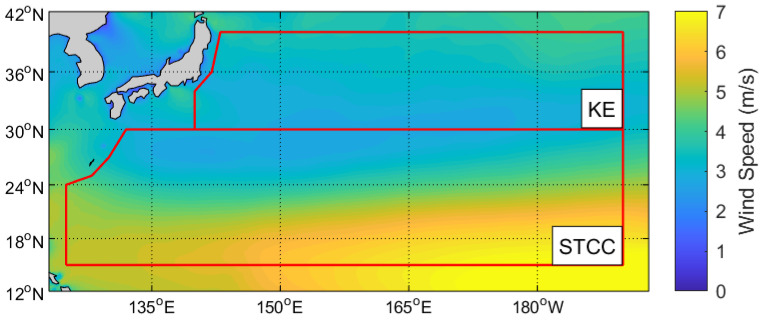
Spatial distribution of multi-annual average sea surface wind speed in the western North Pacific. The red boxes mark the KE and STCC regions. Color bars show units: m s^−1^.

Figure 7 shows that in the low-latitude southern region where the STCC is located, the sea surface wind speed is generally higher, mostly ranging from 5 to 7 m/s. Moving poleward, wind speed decreases gradually, and in the KE region, the climatological wind speed is mostly between 2 and 4 m/s. Notably, this meridional distribution pattern of sea surface wind speed is highly consistent with the observed latitudinal variation in SSTA damping rate, which also increases from high latitudes toward low latitudes. This consistent spatial co-variation provides preliminary observational evidence supporting the regulatory effect of sea surface wind on SSTA damping rate. To quantitatively verify this qualitative inference, we further conducted a linear correlation analysis between damping rate and wind speed to further explore the underlying statistical relationship between the two variables.

**Figure 8:**
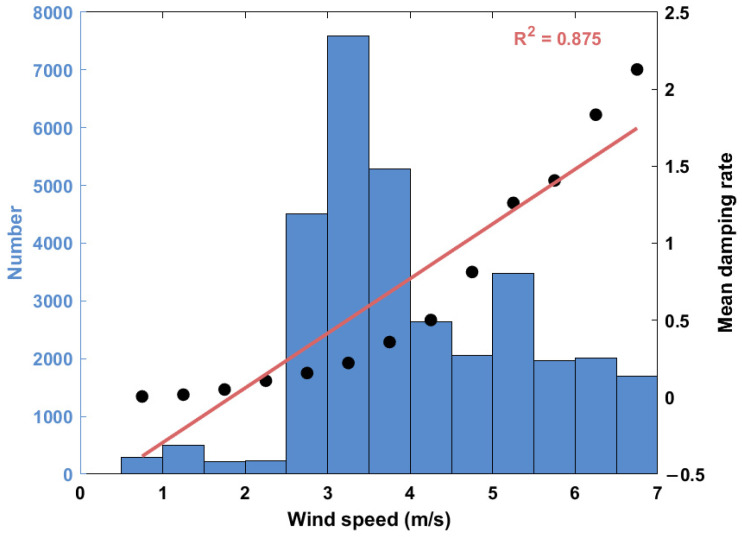
Variation in the domain-averaged normalized damping rate as a function of background wind speed. Background bar plots indicate the number of eddies counted in each bin along the *x*-axis. The red solid line denotes the linear regression fit, and R^2^ indicates the coefficient of determination.

Consistent with the grouped analysis method for heat flux response rate, this study divides the near-surface wind speed into 14 consecutive intervals, ranging from 0 to 7 m/s and an interval step of 0.5 m/s, and then performs the same grouped statistical average processing. The results are shown in Figure 8. It can be seen from the figure that when the wind speed is lower than 3 m/s, the normalized damping rate $\lambda^{*}$ does not change significantly with the increase in wind speed, and it is maintained at a low level on the whole; however, when the wind speed exceeds 3 m/s, $\lambda^{*}$ increases rapidly and approximately linearly with the increase in wind speed. Overall, it shows a significant positive linear relationship. The coefficient of determination of the linear fitting between wind speed and $\lambda^{*}$ is about 0.875, which suggests that the fitting effect is excellent and strongly confirms that the SSTA damping rate does increase significantly with the increase in wind speed. Previous studies on the regulation of eddy-induced SSTA by wind mostly focused on the mechanism of wind-induced upwelling or downwelling, that is, changing the vertical transport of temperature in the upper ocean by adjusting the horizontal divergence of ocean flow, and then regulating the eddy-induced SSTA pattern [42]. This study suggests an alternative pathway: wind speed modulates the SSTA damping rate by influencing the response rate of air–sea turbulent heat flux, and finally affects the formation and evolution process of the eddy-induced SSTA pattern.

## 4. Discussion

This study examines the role of air–sea turbulent heat flux damping in modulating eddy-induced SSTA patterns in the STCC and KE regions of the northwestern Pacific. The composite analysis shows clear regional, seasonal, and polarity-dependent differences in SSTA pattern. The STCC region is characterized by a stronger dipole SSTA pattern, whereas the KE region exhibits a relatively stronger monopole component, particularly for cyclonic eddies. These differences are consistent with the regional contrast in normalized SSTA damping rate.

The results support the interpretation that stronger turbulent heat flux damping tends to suppress the monopole component of eddy-induced SSTA and increase the relative importance of the dipole-asymmetric component. This mechanism is consistent with the stirring–damping framework, in which the observed SSTA pattern reflects a balance between eddy-induced temperature generation, lateral stirring, and air–sea damping. In regions with stronger damping, locally generated SSTAs are damped more rapidly, and the remaining SSTA pattern becomes more strongly influenced by lateral stirring and background SST gradients. In contrast, weaker damping allows the monopole SSTA signal associated with eddy-core temperature anomalies and vertical displacement to persist.

The spatial distribution of the normalized damping rate is more similar to that of the turbulent heat flux response rate than to that of mixed-layer depth. This suggests that the turbulent heat flux response rate is the primary contributor to the large-scale spatial pattern of the damping rate in the present study region. However, mixed-layer depth remains physically important because it controls the heat capacity of the upper ocean and modulates the magnitude of the damping rate. The relatively deep mixed layer in the KE during the cold season may partly offset the effect of enhanced wintertime heat flux response, whereas the shallower mixed layer in the STCC may favor stronger effective damping.

The analysis further suggests that background wind speed can influence eddy-induced SSTA patterns by modulating turbulent heat flux feedback. Stronger winds enhance sensible and latent heat flux responses and thereby increase the effective damping rate. This provides a complementary mechanism to previously emphasized wind–eddy interaction processes, such as wind-induced Ekman pumping and eddy-driven upwelling or downwelling. However, because wind speed directly enters the bulk flux formulation, the relationship between wind speed and damping rate should be interpreted cautiously. It indicates a physically meaningful pathway within the turbulent heat flux feedback framework, but it does not by itself establish an independent causal relationship.

Several limitations should be noted. First, the residual component obtained after removing the azimuthal mean SSTA is interpreted as a dipole-asymmetric component, but it may also include higher-order asymmetries and sampling noise. Second, the present analysis focuses on regional and seasonal composites and does not fully separate the effects of background SST gradients, eddy amplitude, eddy propagation speed, and vertical pumping. Third, damping rates were not separately estimated for individual cyclonic and anticyclonic eddies, which limits our ability to explain polarity-dependent differences in SSTA patterns.

Future studies can apply the latest methods of spatio-temporal pattern mining of dynamic flow data of moving objects to extract eddy characteristics [43], which helps combine turbulent heat flux damping with background SST gradients and eddy dynamical parameters in a multivariate framework to further quantify their relative contributions. The present study does not imply that turbulent heat flux damping is the only factor controlling eddy-induced SSTA patterns. Instead, damping should be considered together with background SST gradients, eddy stirring, vertical pumping, eddy polarity, and mixed-layer processes. Otherwise, our present work focuses on the climatological mean state of eddy-induced SSTA patterns, while the long-term enhancement of eddy heat transport reported in previous studies may cause non-negligible interdecadal modulation on the observed SSTA patterns [44]. This potential long-term linkage deserves targeted quantitative investigation in our follow-up independent research.

## 5. Conclusions

This study investigates the role of air–sea turbulent heat flux damping in shaping eddy-induced SSTA patterns in the STCC and KE regions. The main conclusions are as follows:

First, eddy-induced SSTA patterns exhibit clear regional and polarity-dependent differences. The STCC shows stronger dipole-like structures, whereas the KE is more monopole-dominated.

Second, the STCC exhibits higher normalized damping rates than the KE, consistent with stronger dipole contributions. This suggests that stronger damping tends to favor dipole-like SSTA structures.

Third, the spatial pattern of damping rate is more closely related to turbulent heat flux response than to mixed-layer depth.

Finally, wind speed may influence SSTA patterns by modulating turbulent heat flux feedback, although this relationship is not independent of the bulk flux formulation.

## Data Availability

The mesoscale eddy dataset utilized in this study is the Version 4 eddy product, which was originally developed based on earlier generation eddy datasets (https://aviso.altimetry.fr/, accessed on 29 March 2024). Air–sea interaction variables are obtained from the ERA5 reanalysis product produced by ECMWF (https://cds.climate.copernicus.eu/, accessed on 25 September 2025). The ocean mixed-layer depth dataset is available from the climatological product (https://mixedlayer.ucsd.edu/, accessed on 25 September 2025). Sea surface temperature data are available from CMEMS (https://www.copernicus.eu/en/services/marine/, accessed on 29 March 2024).

## Abbreviations

The following abbreviations are used in this manuscript:

SST	Sea surface temperature
SSTA	Sea surface temperature anomaly
STCC	Subtropical Countercurrent
KE	Kuroshio Extension
CLS	Collecte Localisation Satellites
DUACS	Data Unification and Altimeter Combination System
AVISO	Archiving, Validation and Interpretation of Satellite Oceanographic Data
ECMWF	European Centre for Medium-Range Weather Forecasts
OSTIA	Operational Sea Surface Temperature and Sea Ice Analysis
